# Family History of Diabetes: Exploring Perceptions of People at Risk in the Netherlands

**Published:** 2009-03-15

**Authors:** Miranda Pijl, Lidewij Henneman, Liesbeth Claassen, Danielle R.M. Timmermans, Symone B. Detmar, Giel Nijpels

**Affiliations:** Department of Public and Occupational Health, EMGO Institute, VU University Medical Center; Department of Public and Occupational Health, EMGO Institute, VU University Medical Center, Amsterdam, the Netherlands; Department of Public and Occupational Health, EMGO Institute, VU University Medical Center, Amsterdam, the Netherlands; Department of Public and Occupational Health, EMGO Institute, VU University Medical Center, Amsterdam, the Netherlands; TNO Quality of Life, Leiden, the Netherlands; Department of General Practice, EMGO Institute, VU University Medical Center, Amsterdam, the Netherlands

## Abstract

**Introduction:**

The aim of this study was to explore the perceptions of causes, risk, and control with regard to diabetes and the role of family history among people at increased risk for type 2 diabetes.

**Methods:**

Semistructured interviews were conducted among people aged 57 to 72 years with (n = 9) and without (n = 12) a family history of diabetes.

**Results:**

Participants mentioned different causes for diabetes; these were often a combination of genetic and behavioral factors. Some participants with a family history expressed incoherent causal beliefs; their general ideas about the causes of diabetes did not explain why their relatives were affected. The role of genetics as a cause for diabetes was more pronounced when people perceived diabetes as "running in the family," and this finding did not necessarily relate to a high number of affected relatives. Although people with a family history were aware of the diabetes in their family, they did not always associate their family history with increased risk, nor did they worry about getting diabetes. The absence of diabetes in the family was often used as a reason to perceive a low risk. Participants who primarily perceived genetic predisposition as a cause felt less able to prevent getting diabetes.

**Conclusion:**

Future diabetes prevention strategies would benefit from giving more attention to individual perceptions, especially in the context of family history, explaining the multifactorial character of diabetes, and highlighting effective ways to reduce the risk.

## Introduction

The prevalence of type 2 diabetes mellitus, a serious health problem, is increasing. Several factors contribute to this increase, including increasing obesity and inactivity (1). In high-risk people, the onset of and complications from type 2 diabetes can be delayed or even prevented by adopting a healthy lifestyle (2). Prevention is especially important for people with a family history of diabetes because a family history is one of the strongest risk factors for type 2 diabetes (3-5). Studies consistently report a 2- to 6-fold increased risk of diabetes associated with a positive family history, depending on the number and closeness of relatives affected (6,7). Family history represents genetic, environmental, and behavioral elements, and the interactions between them.

Despite the high prevalence of type 2 diabetes, little is known about the perceptions of diabetes risk among high-risk populations. Quantitative studies show that people with a family history of type 2 diabetes perceive a higher risk of getting diabetes compared with those at average risk (8), but they still often underestimate their actual risk (9). Furthermore, only about half of these people believe that diabetes is preventable (10,11). Walter and Emery (12) have shown that in comparing perceptions of people with a family history of diabetes, cancer, or heart disease, diabetes was generally seen as the least threatening. More in-depth information about these perceptions of people with a positive family history of diabetes is needed so that effective targeted prevention strategies can be designed.

Although family history is not a modifiable factor, communicating familial risk information may be useful in raising risk awareness, thereby encouraging preventive behaviors (13). At the same time, familial risk information may result in a sense of fatalism if people see familial risks as deterministic, thereby discouraging healthy behavior. According to Marteau and Weinman (14), understanding the conditions under which genetic risk information does and does not motivate behavior change is the first step toward developing ways of communicating such information to maximize its motivational impact. Perceptions about genetic risk are thought to be mainly influenced by causal beliefs, since genetic information is specifically about causes (15). In addition, both risk perception (threat appraisal) and perceptions of control (coping appraisal) can be used to explain people's motivation to improve their lifestyle to reduce their risk for type 2 diabetes (16).

The aim of this study is to explore causal beliefs, perceived risk, and perceptions of control among people at increased risk for getting diabetes, and the role of family history in this context. Perceptions of people with and without a family history are explored to compare and contrast these beliefs between groups.

## Methods

### Participants

A sample was recruited from a database of a population-based targeted diabetes screening study that was carried out from 1998 to 2000 among inhabitants of the West-Friesland region of the Netherlands (for details, see Spijkerman et al [17]). Participants (N = 2,315) had been at increased risk of developing diabetes on the basis of a self-reported risk questionnaire. However, blood test results excluded them from having developed the disease at that time. The participants had been informed by letter that they did not have diabetes, but no further information was provided. For our study, people older than 75 years were excluded; of all eligible people, 20 people with at least 1 first-degree relative with diabetes and 20 people without a family history of diabetes were randomly selected on the basis of self-reported information.

A municipal official checked the 40 addresses to determine whether people had moved or died. Two people had died and 1 had moved, leaving 37 people who were sent a letter of invitation signed by a general practitioner. Exclusion criteria for the interview study were not speaking the Dutch language and having been told that they had diabetes. In total, 31 of 37 people (84%) responded to the invitation (14 were sent a reminder), and 24 people (65%) agreed to participate. Of the nonparticipants, 1 reported having developed type 2 diabetes; 1 participant was on vacation during the study period; and 5 others did not want to participate and gave no reason for nonparticipation. In the analyses, 3 participants were excluded because they appeared to have only second-degree relatives with diabetes, leaving only 9 people with a positive family history and 12 people without a positive family history.

### Methods

The 3 core concepts in this qualitative study — causal beliefs, risk perception, and perceptions of control — were used to construct a semistructured interview guide. Additional themes were participants' personal family history of diabetes and perceived consequences of diabetes. Two researchers (M.P. and L.C.) conducted the interviews in the participants' homes. Interviews were held in June 2005 and lasted 30 to 60 minutes. The interview guide was refined after the first 2 interviews, following discussion among 4 researchers (M.P., L.H., L.C., and D.T.) using the taped interviews. The Medical Ethical Committee of the Vrije Universiteit University Medical Center approved the study, and every participant signed an informed consent form before participation.

### Analysis

All interviews were audiotaped and transcribed. Content analyses were conducted on the transcripts (18). On the basis of found codings, further analyses were conducted to detect correspondence and differences between people with and without a family history. Coding was subsequently completed by 2 of the authors (M.P. and L.H.). To ensure uniform coding, the 2 authors coded each transcript and then discussed the codings until agreement was reached. For the analysis, we used Kwalitan version 5.0 (Department of Research Methodology, Radboud University, Nijmegen, the Netherlands). The most important themes are presented, and quotations are used to illustrate the meanings that participants attached to a theme. Participants' identification number (#), age in years, sex, and family history (FH) are presented. Perceptions of people without a family history are used to compare and contrast the findings among people with a family history and are not described in detail.

## Results

The characteristics of the participants with (n = 9) and participants without (n = 12) a family history of diabetes who were interviewed are shown in the Table. Participants with a family history of diabetes reported 1 to 4 affected relatives among first- and second-degree relatives. The mean age in the group of people with a positive family history was 67 years (range 62-72), and for the group without a family history was 66 years (range 57-71). Participants varied in educational level, and in both groups approximately a quarter of the participants were highly educated.

### Causal beliefs

**Both genetic and behavioral causes**

Participants in both groups were often able to name several causes for diabetes, including genetic and behavioral causes. Causes mentioned were genetic predisposition (including family history), unhealthy food (too much fat and sugar, unvaried diet), lack of physical activity, stress, alcohol intake, and age. Participants often mentioned genetic predisposition as a cause of diabetes in combination with an unhealthy lifestyle, whether they had a family history or not. For example, this man said:

I think [diabetes] has to do with eating habits, if I understood it correctly. But I think that it's also a hereditary matter, that someone inherits it. That the mother or father possibly had it. (#131, 59, M, no FH)

**General ideas about causes do not explain diabetes in the family**

Compared with people without a family history, those with a family history sometimes expressed less coherent thoughts about the causes of diabetes. The following quotation is from a woman who had explained earlier in the interview that she predominantly saw genetic predisposition as a cause of diabetes, though when she explained why her relatives developed diabetes, she had another perception:

I think that it's always genetically determined whether you get it. . . . I think that for my mother it was caused by stress, when my father died. . . . For my father, it was his lifestyle. I think that the diabetes that my father had wasn't hereditary for us. Because my father too was always busy. (#106, 64, F, FH: father, mother, mother's sister)

Others who generally perceived an unhealthy lifestyle (eg, unhealthy diet, overweight) to be the cause of diabetes showed confusion about the cause of the diabetes in their family when lifestyle could not be seen as an explanation for their affected relative. For example, this man commented:

People with overweight have a high risk of getting diabetes. It has to do with food. . . . My brother has had diabetes since years. He's very slim, and he was also skinny when he got it, so that's miraculous. Yes, in this case [heredity] could play a role. (#107; 67; M; FH: brother)

**Diabetes runs in my family, thus genetic cause**

For people with a family history of diabetes, the role of genetics as a cause for diabetes was seen as more pronounced when people perceived diabetes as "running in the family," particularly when diabetes was passed on from generation to generation in the same lineage. For example, this woman has a family that is heavily affected with diabetes, and therefore she believed that she has inherited the predisposition for diabetes:

Diabetes runs in our family, because my old grandmother suffered from it to a lesser extent, my father had it very severely, my sister injects, and my brother controls it with medication. So then you do think there's something inside you. (#115; 65; F; FH: mother's father, father, brother, sister)

However, this phenomenon also occurred when only 1 relative was affected, as in the following example of a woman who mentioned that diabetes has a genetic cause:

So I think it's just genetic, that you can't prevent it. My risk is somewhat higher [than that of a random man or woman of the same age], because I have diabetes in the family. (#118; 62; F; FH: mother)

**Behavior triggers the course of diabetes**

Although some believed that behavioral factors, such as an unhealthy diet, were not the cause of diabetes, they thought that these factors might influence the course of the disease (ie, causing an earlier onset of diabetes or more severe symptoms) in case of genetic predisposition. The following quotation from a woman illustrates this perception:

I think it's in your genes. I don't think if you eat too many sweets that you get [diabetes]. You will just get fat. Well, if you have diabetes you shouldn't eat sweets. It maybe just makes [the diabetes] worse. (#118; 62; F; FH: mother)

**"Inherited lifestyle"**

One participant believed that the diabetes in his family was caused by an "inherited lifestyle," in particular their diet:

My sister's lifestyle is sloppy, that might be the cause [of her diabetes]. Being rather overweight, quite a lot of food, never exercising. [As to the cause of diabetes for my brother,] the only possible thing is that he has the same lifestyle as my sister. Eating a lot of sweets and a lot of food, lots of fat. That might be an inherited factor. (#132; 64; M; FH: brother, sister)

### Perceived risk

**Diabetes in my family, therefore increased risk**

Only 4 of 9 participants with a positive family history perceived a slightly higher risk when comparing themselves with other people of the same age, because of the diabetes in their family.

Maybe I have a higher risk [of getting diabetes] because my mother had diabetes. (#108, 69, F, FH: mother, mother's mother)

**Diabetes in my family, but no risk for me**

Although participants with a family history did mention that they had diabetes in their family, they did not always seem to associate this information with their own risk. For example, this woman said:

The risk of getting diabetes is on my mind. I do have a mother and a grandmother who had it. But [my chance of getting diabetes] is the same, everybody can get it, I don't think my risk is higher or lower. (#113; 71; F; FH: mother, mother's mother)

**Diabetes not in my family, so no risk for me**

Most of the participants without a family history perceived a low risk of getting diabetes. Only a few (3/12) perceived themselves at a slightly higher risk than average because they considered themselves to be overweight and to have an unhealthy lifestyle. Moreover, the absence of diabetes in the family was often (7/12) mentioned as a reason to perceive a low diabetes risk. For example, this woman:

The [diabetes] risk must be very low, because at home there were 7 of us, and none of us has it! (#130; 71; F; no FH)

**Despite high risk-awareness, low emotional response**

Participants who did mention severe consequences of diabetes and sometimes even perceived a high risk due to an extensive number of affected family members still did not worry about getting diabetes. The following quotation is from a woman who mentioned in the interview that she perceived a high risk of getting diabetes:

Blindness, the legs, the muscles or nerves in the legs of my father were affected. . . . No severe illness, they say you can become 100 years old with it, but I think the side effects are very hard. I never think about getting diabetes. [Having diabetes] is not a problem; I mean, you just pay more attention to what you eat. (#115; 65; F; FH: mother's father, father, brother, sister)

### Perceptions of control

Although some people correctly stated that having a healthy diet and being physically active can delay the onset of or even prevent diabetes, most participants in both groups were unaware of ways to prevent diabetes. Participants with a family history held different beliefs about ways to control their risk, depending on their causal beliefs.

**Genetic cause, cannot control risk**

Participants with a family history of diabetes who mainly perceived genetic causes for getting diabetes all felt that they were not able to prevent it. At most, they thought they might be able to postpone the disease by adopting a healthy lifestyle, like this woman:

I think that it's always genetically determined whether you get [diabetes]. I think there's little you can do about it then. You might be able to postpone it a little, if you know you can get it, by paying attention to what you eat. (#106; 64; F; FH: father, mother, mother's sister)

**Behavioral cause, can control risk**

In contrast, those with a family history who predominantly saw lifestyle as a cause of diabetes did believe there were ways to prevent diabetes, as this man's comments illustrate:

Of course you always have an influence [on the chance of getting diabetes] by not doing things that can cause diabetes . . . like having lots of fat and drinking sweet cola. (#107; 67; M; FH: brother)

## Discussion

Both people with and without a family history of diabetes mentioned several causes for diabetes, including genetic and behavioral causes. This finding suggests that people correctly see diabetes as a multifactorial disease. Walter and Emery (19) earlier described the multifactorial model of familial disease risk. They also showed that people at risk for diabetes view lifestyle factors as triggering an underlying risk, for example, genetic risk. In our study, some participants thought that lifestyle could worsen the diabetes or that it would develop sooner in case of genetic predisposition. None of them mentioned that an unhealthy lifestyle alone would trigger an underlying risk. One participant, however, pointed out that lifestyle might be an inherited factor. This may imply that some people identify behavior as heritable. Because only 1 participant mentioned this aspect, it is difficult to draw such a conclusion.

A contrast between both groups concerning causal beliefs was that people with a family history of diabetes were less coherent and more confused when talking about the causes of diabetes, since their general beliefs about the causes did not always explain why their relatives were affected. Being incoherent about causal beliefs has been identified previously for diabetes patients. Though aware of possible risk factors for diabetes (eg, being overweight, physically inactive, having a family history), patients without these risk factors could not understand how they had developed the disease (20). Thus, their general ideas about the causes of diabetes did not explain why they were affected. It seems that people have difficulty in understanding the interplay between genes and environment or behavior (eg, unhealthy lifestyle). Possibly people have more difficulty in integrating risk information from more than 1 source than when there is a single risk factor for a disease, as Marteau and Weinman have suggested (14).

People mostly perceived a genetic cause for getting diabetes when they perceived diabetes as "running in the family." This belief was reported when several affected relatives were of the same lineage, as one might expect. However, a woman with only 1 affected relative also perceived diabetes as running in the family and perceived a higher risk of getting diabetes. In a quantitative study designed to identify determinants of familial risk perception of common diseases (cancer, coronary heart disease, and diabetes), Walter et al (21) found that believing the disease "runs in the family" is an important predictor of perceiving a familial risk, together with believing the disease has a genetic cause and diabetes is a serious condition. People without a family history often see not having a family history as protective, which might indicate that they also perceive diabetes is caused by a genetic predisposition when it runs in the family.

Some people who saw diabetes as running in the family indeed perceived an increased risk of getting diabetes because of the occurrence of diabetes in the family. This finding might indicate that people associate their beliefs about heredity of diabetes with their perception of being at risk. Participants with a family history perceiving behavioral causes for getting diabetes had a different risk perception. Though they acknowledged their family history, when comparing themselves with other people of the same age, they still did not perceive a higher risk. In line with these findings, Harrison et al (22) found that less than 40% of the people with a family history of diabetes perceived themselves to be at increased risk. Despite their family history of diabetes, participants in this study did not feel worried about getting it. Walter and Emery (12) earlier described that diabetes was not viewed as a serious disease but as a chronic disease of older age and at worst a minor inconvenience. In keeping with these findings, Eborall et al (23) described low levels of anxiety among participants of a screening program for type 2 diabetes in the east of England, even those eventually diagnosed with type 2 diabetes.

This study suggests that people with a positive family history, especially people who perceive genetic causes for getting diabetes, are less likely than others to believe that diabetes is preventable, supporting the results of Harwell et al (10). In contrast, it seemed that people who perceived behavioral causes for developing diabetes did believe that diabetes was preventable. A previous study by Senior et al (24), considering perceptions about an inherited predisposition to heart disease (familial hypercholestrolemia), showed evidence for fatalistic beliefs when the underlying cause of a positive test result was seen as genetic, and no such evidence was found for perceptions of underlying behavioral causes.

Some limitations of our study need to be addressed. Participants were older adults who might consider health risks a part of getting older, may be less engaged in preventing disease, and may have had fewer concerns about getting premature disease due to their family history. The participants had been in a stepwise-based screening study some years earlier; therefore, their knowledge about diabetes might have been better than that of the average population. Nevertheless, these results suggest that, even in this group, knowledge about diabetes and especially ways to prevent it were suboptimal. Although the small sample size of the study may limit conclusions, we have gained a deeper understanding of the perceptions and beliefs of people with a family history of diabetes.

This study suggests that people probably used causal beliefs to construct their perceptions of risk and control (ie, when people perceive genetic causes for diabetes, they tend to have a higher perception of risk and lower perception of control). Perceptions of risk and control in turn may be important motivating factors for preventive health behaviors (16). People with a family history of diabetes seem to have incoherent causal beliefs; therefore, prevention programs should promote correct understanding of the multifactorial causes of type 2 diabetes among people at high risk due to their family history, which might have a positive effect on their perceptions of risk and control and, directly or indirectly, on preventive behaviors. In addition, people without a family history would also benefit from clear information on this topic.

The findings of the study point to the need for more research on this topic, for example, on the relationship between causal beliefs and both risk perception and perception of control.

## Figures and Tables

**Table.** Participant Characteristics, Semistructured Interviews on Perceptions of People at Familial Risk of Diabetes in the Netherlands, 2005

Participant #^a^	Age, y	**Sex**	**Relative With Diabetes**
101	70	Male	None
105	70	Female	None
106	64	Female	Father, mother, mother's sister
107	67	Male	Brother
108	69	Female	Mother's mother, mother
112	69	Female	None
113	71	Female	Mother's mother, mother
115	65	Female	Mother's father, father, brother, sister
117	69	Male	None
118	62	Female	Mother
119	57	Male	None
121	57	Female	None
124	71	Female	None
127	70	Male	None
129	71	Male	None
130	71	Female	None
131	59	Male	None
132	64	Male	Brother, sister
133	68	Female	Mother
134	61	Female	None
136	72	Male	Mother, daughter of sister

a All people who were eligible to participate in the study were numbered. Only numbers of participants included in the article are listed in the table, so the numbers are not sequential.
